# Relationship Between Emotional Labor and Job Performance in Emergency Nurses: Multiple Mediating Effects of Caring Ability and Communication Competency

**DOI:** 10.1155/jonm/2258686

**Published:** 2026-06-12

**Authors:** Danni Feng, Sufang Huang, Jie Xiong, Jing Cheng, Jie Fu, Yaru Xiao, Danli Zheng

**Affiliations:** ^1^ Department of Nursing, Tongji Hospital, Tongji Medical College, Huazhong University of Science and Technology, Wuhan, 430030, Hubei, China, hust.edu.cn; ^2^ School of Nursing, Tongji Medical College, Huazhong University of Science and Technology, Wuhan, 430030, Hubei, China, hust.edu.cn; ^3^ Department of Emergency, Tongji Hospital, Tongji Medical College, Huazhong University of Science and Technology, Wuhan, 430030, Hubei, China, hust.edu.cn

**Keywords:** caring ability, communication competence, emergency nurses, emotional labor, job performance

## Abstract

**Aim:**

Emergency nurses face tough occupational challenges and substantial emotional burdens, which can impact their job performance. Caring behavior and communication are essential components of patient‐centered care, but their role in the relationship between emotional labor and job performance is unclear. This study aimed to explore the mediating role of caring ability and communication competency on emotional labor and job performance.

**Design:**

Cross‐sectional study.

**Methods:**

A cross‐sectional study of 210 emergency nurses was conducted at eight city tertiary hospitals in central, eastern, and southwestern China from November 2023 to January 2024. Potential participants were selected using a convenience sampling method. The study variables, including emotional labor, caring ability, communication competency, and job performance, were assessed via self‐report questionnaires. Path analysis using a multiple mediation model was performed using AMOS 28.0.

**Results:**

The results of this study showed that the mean score of emergency nurses’ job performance score was 4.53 ± 0.52. Caring ability and communication competence partially mediated the relationship between deep acting and job performance, with effect sizes of 0.022 and 0.100, respectively. The chain‐mediating effect of caring ability and communication competence was 0.071.

**Conclusion:**

Emotional labor did not directly influence job performance. The emotional management strategy of deep acting was the sole approach that indirectly influenced job performance via the complete mediating role of caring ability and communication competence. Emergency nurses utilizing deep acting tactics can more easily identify and regulate their emotions, exhibit greater empathy, treat patients sincerely, communicate effectively, and improve job performance.

**Impact:**

Nursing managers and educators should attach importance to the emotional labor of emergency nurses, particularly cultivating and fostering deep acting, and take measures to strengthen communication skills and caring education to improve job performance and provide better nursing services for patients.

**Patient or public contribution:**

No patient or public contribution.

## 1. Background

Emergency nursing work is at the forefront of healthcare services in the rapidly developing healthcare system. It is responsible for saving lives and alleviating pain while facing enormous work challenges and heavy emotional burdens [1, 2]. Health services are evolving from disease‐centered care to patient‐centered care, with patients actively participating in health services [3]. Emergency nurses must possess superior professional skills to cope with rapidly changing conditions, maintain keen emotional insight, and communicate with patients and their families with empathy. Emotional labor becomes an integral part of this process. The emergency care environment in China is markedly unique, as the high intensity of work, the risk of occupational violence, and the shortage of human resources put emergency nurses under tremendous pressure in their daily work [4, 5]. Moreover, in the emergency department, medical staff face immense time pressure and must quickly make efficient decisions and handle patients [6]. All these factors have burdened emergency nurses in China, leading to increased role overload [7]. Resource conservation theory shows that role overload can increase psychological stress [8]. When individuals are in a state of high pressure for a long time, their mental health is often severely affected, which is manifested by an increase in negative emotions such as anxiety and depression [9]. These emotions both affect an individual’s mental health and become a potential threat to job performance. Specifically, negative feelings will cause an individual to be unable to concentrate, reduce the efficiency and accuracy of decision‐making, and have a direct negative impact on their job performance [10, 11]. Given the above background, this study focused on the complex relationship between emotional labor and the job performance of emergency nurses in China from the perspective of emotional burden, especially by introducing caring ability and communication competency as multiple mediating variables and exploring the mechanisms of this relationship in depth. It aimed to provide a scientific basis and practical guidance for enhancing the job performance of emergency nurses, alleviating their psychological pressure, and promoting the continuous improvement of medical services.

### 1.1. The Influence of Emotional Labor on Job Performance

Emotional labor is how nurses display facial and physical states that meet organizational requirements in front of patients by regulating their emotions [12]. Nursing is highly emotional labor involved and committed work. Emotional labor is divided into three emotion regulation strategies: surface acting, emotional expression requirements, and deep acting [13]. Given the distinct outcomes of these three strategies, they should be analyzed separately. Surface acting refers to nurses’ outward displays of emotions to present nonauthentic emotional feelings that meet the demands of their roles. This strategy usually involves simply suppressing genuine emotions to fulfill the job’s requirements. For example, a nurse may force herself to smile and show caring when dealing with a patient in pain, even if internally she feels frustrated or helpless. Although surface acting can effectively meet job demands in the short term, long‐term use may lead to emotional exhaustion and professional burnout [14]. Emotional expression requirements refer to organizational regulations on the emotions exhibited by employees when interacting with clients. These requirements are frequently based on workplace norms and customer expectations. Studies have demonstrated that moderate emotional expression requirements can help nurses express their affection and care for patients, respond with pleasant emotions, and be identified, rewarded, and promoted by the organization. However, if emotional expression standards are overly rigorous, employees may feel emotional exhaustion, which can decrease job performance [15, 16]. Deep acting refers to nurses actively adjusting speech, body movements, facial expressions, and other behaviors to align their interior feelings with their external performance. Deep acting can express empathy and care for patients, encourage nurses to stimulate positive emotions, maintain an engaged psychological state, and improve job satisfaction and performance [17]. Therefore, we hypothesize the following.

**Hypothesis 1.**

Emotional labor will significantly predict job performance (emotional labor ⟶ job performance).

### 1.2. The Mediating Role of Caring Ability Between Emotional Labor and Job Performance

The core value of nursing is humanistic care. Caring in nursing was also expressed as an intrinsic feeling integrating the ethics of nursing and professional caring [18]. Caring ability denotes a nurse’s capacity to manifest their intrinsic caring spirituality in the delivery of patient care [19]. The ability to care is crucial for nurses in the emergency department. Nurses possessing caring competencies typically execute nursing tasks more effectively. They are more inclined to cultivate positive relationships with patients and receive favorable feedback, which enhances their job satisfaction and subsequently promotes greater job engagement and performance among nurses [20]. Caring competence is a crucial aspect of professionalism for emergency nurses. By consistently enhancing their caring competence, nurses can demonstrate improved job performance, attain more career accomplishments, and deliver high‐quality patient care [21]. Emotional labor influences the quality of humanistic care nurses provide; increased utilization of deep acting tactics enhances their ability to foster empathy with patients and improves the quality of humanistic care, but surface acting plays the opposite role [15]. Consequently, we hypothesize the following.

**Hypothesis 2.**

Emotional labor will influence job performance through the mediating effect of caring ability (emotional labor ⟶ caring ability ⟶ job performance).

### 1.3. The Mediating Effect of Communication Competency Between Emotional Labor and Job Performance

Communication constitutes exchanging information, emotions, and comprehension, essentially centered on efficiently conveying information and shared understanding. In nursing, communication is the link for transmitting medical information and comprehending patient needs while also being essential for fostering trust, strengthening understanding, improving treatment outcomes, and increasing patient satisfaction [22, 23]. Effective communication can improve nurses’ work performance by facilitating a better knowledge of patient needs, delivering customized nursing care, minimizing misunderstandings and disputes, fostering teamwork, and enhancing work efficiency and patient satisfaction [24, 25]. Emotional labor, a vital nursing component, is closely linked to communication [26]. The empathy, patience, and care nurses exhibit in emotional labor are expressed through effective communication, strengthening the emotional bond between nurses and patients and enhancing the nursing quality and patient experience [27]. Therefore, we propose the following hypothesis.

**Hypothesis 3.**

Emotional labor will influence job performance through the mediating effect of communication competency (emotional labor ⟶ communication competency ⟶ job performance).

### 1.4. The Chain‐Mediating Effect of Caring Ability and Communication Competency on Emotional Labor and Job Performance

A good nurse–patient relationship is essential for enhancing nursing quality and patient satisfaction. The foundation of this relationship is built on trust, communication, empathy, and active listening. Nurses can cultivate trust with patients, relieve their worry and apprehension, and promote effective communication by exhibiting caring behaviors [28]. Studies indicate that patients who trust the nursing team experience a sense of safety, support, and confidence in the treatment process, resulting in enhanced patient satisfaction, superior treatment outcomes, and improved nurse performance [29].

Consequently, we put up the subsequent hypothesis.

**Hypothesis 4.**

Caring ability and communication competency will jointly play an intermediary role in the relationship between emotional labor and job performance (emotional labor ⟶ caring ability ⟶ communication competency ⟶ job performance).

The literature review revealed a lack of inductive research examining the relationship between emotional labor and job performance among emergency department nurses. Furthermore, prior research has explored the mediating influence of nurse–patient trust and patient‐centered nursing on the relationship between emotional labor and job performance [30]. Caring behavior and communication are essential components of patient‐centered care [31], yet mechanisms linking emotional labor and job performance remain unclear. Therefore, this study explored the mechanisms underlying the relationship between emotional labor, caring ability, communication competence, and job performance, aiming to provide suggestions for effectively enhancing emergency nurses’ job performance to improve overall nursing quality.

## 2. Methods

### 2.1. Study Design, Settings, and Population

The study adopted a descriptive cross‐sectional design and was conducted in line with the Strengthening the Reporting of Observational Studies in Epidemiology (STROBE) guidelines [32]. A convenience sampling method was applied to recruit emergency nurses from 8 city tertiary hospitals in central, eastern, and southwestern China from November 2023 to January 2024. The inclusion criteria for the study were as follows: (1) emergency nurses obtaining a professional qualification certificate, (2) age ≥ 18 years old, and (3) working in this hospital for more than six months. Exclusion criteria were as follows: (1) work content does not directly contact the patients and family members, (2) vacation time > 1 month, (3) not willing to cooperate with the investigation, and (4) nurse trainees or assistant nurses.

### 2.2. Measurements

#### 2.2.1. Sociodemographic Variables

According to the purpose of the research, a self‐designed general demographic questionnaire was adopted, including age, gender, nursing years, education, and professional titles.

#### 2.2.2. Emotional Labor Scale (ELS)

Luo and others developed ELS based on the nursing situation in China [33]. This scale is widely used among nurses in China, including three dimensions: surface acting (1–7 items), deep acting (12–14 items), and emotional expression requirements (8–11 items), with a total of 14 items. Each item is scored using a six‐point Likert scale ranging from “strongly disagree” to “strongly agree.” The higher the score, the higher the emotional labor level of the nurse. The Cronbach’s alpha for the entire ELS was 0.811, and the Cronbach’s alpha values for the three factors ranged from 0.711 to 0.872. The Cronbach’s alpha of the scale in this study was 0.935, and the Cronbach’s alpha values for the three factors ranged from 0.900 to 0.938.

#### 2.2.3. Caring Ability Inventory (CAI)

CAI was developed by nursing expert Professor Nkongho in 1990 [34] and translated and revised by Ma in 2012 into a Chinese version [35]. It contained 37 items (including 13 reverse entries) and was classified into three dimensions: the knowing dimension (14 items), which referred to the degree of understanding, grasping, and processing of objective information such as individuals, others, and the surrounding environment; the dimension of courage (13 items) referred to the degree to which one actively cared about oneself and others and dared to face unknown situations; and the dimension of patience (10 items) referred to the ability to endure and persevere with perseverance. According to the Likert 7‐point scoring system, the CAI scale was assigned 1–7 points based on the degree of opposition or agreement, with 1 being “completely opposed” and 7 being “completely agreed.” The Cronbach’s alpha for the entire CAI was 0.925, and the Cronbach’s alpha values for the three factors ranged from 0.816 to 0.929. The Cronbach’s alpha of the scale in this study was 0.851, and the Cronbach’s alpha values for the three factors ranged from 0.851 to 0.940.

#### 2.2.4. The Nurses’ Clinic Communication Competency Scale (NCCCS)

NCCCS was developed and applied by Zeng of Central South University in 2010 [36], which comprehensively evaluated 58 indicators of communication competency between nurses and patients and better reflects the level of clinical communication competency of nurses. This scale included six dimensions [37]: team communication competency (6 items), basic language communication competency (11 items), basic nonlanguage communication competency (7 items), emotional perception competency (9 items), emotional support competency (6 items), and communication competency in difficult clinical scenes (19 items) The Likert 5‐point system scores from 1 point (*very poor*) to 5 points (*very good*), with a total score of 58–290 points. The higher the score, the stronger the nurses’ clinic communication competency. The homogeneity reliability of the whole scale was 0.978, and the coefficients of the six aspects ranged from 0.868 to 0.954. The Cronbach’s alpha of the scale in this study was 0.992, and the Cronbach’s alpha values for the six factors ranged from 0.937 to 0.984.

#### 2.2.5. Job Performance Scale (JPS)

Job performance was measured by the 16‐item Chinese version of the JPS [38], which comprises two subscales: task performance (1–9 items) and contextual performance (10–16 items). Each item is scored using a 5‐point Likert scale ranging from 1 (*strongly disagree*) to 5 (*strongly agree*). The higher the score, the stronger the job performance level. The Cronbach’s alpha values for the two factors ranged from 0.923 to 0.928. The Cronbach’s alpha of the scale in this study was 0.981, and the Cronbach’s alpha values of each factor were 0.967.

### 2.3. Data Collection

This study conducted an anonymous survey in the form of an electronic questionnaire, and the data were strictly confidential. The research team comprises nursing graduate students, managers, and clinical nurses. After determining the collection area, the project leader contacted the management of each hospital and invited emergency department nurses who met the inclusion criteria and agreed to participate to complete the investigation. At the beginning of the questionnaire, the study’s purpose, methods, and significance were explained to the participants, who voluntarily participated and had the right to withdraw at any time. In addition, an informed consent form has been set up, and only when participants click on the consent form can they access the online questionnaire and start filling it out. To avoid “one person, multiple answers,” each device could only be used to answer once. The questionnaire can be exited at any time and temporarily saved to ensure participants can fill it out during breaks. In addition, the research team also prepared some small gifts to encourage participants to participate in the survey.

According to reports in previous research [8], the standard deviation of the job performance of the general population in Chinese nurses was 0.42, the test level a was taken as a bilateral 0.05, and the allowable error was 0.16 (15%). Considering the 20% loss rate, the required sample size was 32. A total of 234 nurses submitted questionnaires, of which 15 were answered by nonemergency department nurses, and 9 were of low quality (all answers were repeated). Finally, a total of 210 nurses (89.7%) were included in the analysis. The formula for calculating the sample size is as follows (*α*: Type I error; *σ*: standard deviation; and *δ*::allowable error):

(1) n= zα2∗σ2δ2.

### 2.4. Statistical Analysis

Continuous variables were presented as mean ± standard deviation or median and interquartile range, depending on whether the variable conformed to a normal distribution. The frequencies and percentages of categorical variables were presented in descriptive statistics. We used SPSS software to judge the distribution (skewness: |Sk| < 3; kurtosis: |Ku| < 10)[39]. The data of ELS, CAI, NCCCS, and JPS presented a normal distribution (skewness < |1.346| and kurtosis < |1.431|). Harman′s single‐factor analysis assessed the common method bias, and Pearson correlation analysis was used to explore the relationships between variables. We employed a structural equation model to identify both direct and indirect relationships in the model. We examined the multivariate normality test. Findings showed that the multivariate kurtosis composite reliability (CR) (32.346) was higher than the recommended value of 5[40]. Due to the lack of multivariate normality, the Bollen–Stine bootstrap method was adopted to adjust the inflated *χ* ^2^ value to improve the overall model fit [41]. A total of 2000 bootstrap samples were made to calculate the mediating effect. The path analysis model based on the assumptions was built using the maximum likelihood estimation method. The measurement model was examined for reliability (Cronbach’s alpha < 0.7, acceptable), convergent validity (average variance extracted [AVE] < 0.5 and CR > 0.6, satisfactory), and discriminant validity (the AVE square root should be larger than the correlations with other latent components, satisfactory) [42, 43]. The absolute fit method was adopted, which featured the following parameters [44, 45]: *χ* ^2^/df (cutoff ≤ 3) and root mean square error approximation (RMSEA) (cutoff < 0.08, good fitting). The cutoff value of ≥ 0.90 of the model fit indices, including the goodness of fit index (GFI), adjusted GFI (AGFI), comparative fit index (CFI), incremental fit index (IFI), and normed fit index (NFI), was considered suitable. Statistical significance was set at *p* < 0.05, and data were analyzed by SPSS Statistics 24.0 (IBM Corporation, Armonk, NY, USA) and Amos 28.0 (IBM, USA).

### 2.5. Ethical Considerations

The study was conducted in accordance with the Declaration of Helsinki [46] and was approved by the Medical Ethics Committee of Tongji Hospital affiliated to Tongji Medical College of Huazhong University of Science and Technology, China (approval number: TJ‐IRB202401086).

## 3. Results

### 3.1. Harman Single‐Factor Analysis

The self‐reported nature of the data meant the possibility of common method bias [47]. The Harman single‐factor analysis showed that the eigenvalues of the 16 common factors were greater than 1. The first common factor explained 43.69% of the variance, lower than the recommended threshold of 50% [48]. Therefore, no common method bias was detected.

### 3.2. Demographic Characteristics

The general characteristics of the 210 emergency nurses are summarized in Table 1.

**TABLE 1:** General characteristics of emergency nurses (*N* = 210).

Characteristics	Categories	*n*	%
Age, y	≤ 30	94	44.8
	31–40	91	43.3
	> 40	25	11.9

Gender	Male	35	16.7
	Female	175	83.3

Nursing years, y	≤ 5	54	25.7
	6–10	80	38.1
	11–15	45	21.4
	> 15	31	14.8

Education	Junior college and below	15	7.1
	College	185	88.1
	Graduate and above	10	4.8

Professional titles	Nurses	35	16.7
	Senior nurse	65	31.0
	Supervisor nurse	98	46.7
	Deputy chief nurse and above	12	5.7

The age range of participants was 20–55 (32.25 ± 6.82) years old.

### 3.3. Descriptive Statistical Analysis of Variables

The study involved four variables: ELS, CAI, NCCCS, and JPS. The sum and mean scores of each variable of the 210 emergency nurses are summarized in Table 2.

**TABLE 2:** The sum scores and mean scores of each variable (*N* = 210).

Variables	Categories (items)	Possible range	Min–max	Sum score (mean ± SD)	Mean score (mean ± SD)	Skewness	Kurtosis	Cronbach’s alpha
ELS	Surface acting	7–42	7–42	25.76 ± 8.99	3.68 ± 1.28	0.133	−0.601	0.936
	Emotional expression requirements	4–24	4–24	15.09 ± 5.32	3.77 ± 1.33	−0.018	−0.632	0.900
	Deep acting	3–18	3–18	14.61 ± 3.13	4.87 ± 1.04	−0.920	0.997	0.938
	Total score	14–84	14–84	55.46 ± 14.79	3.96 ± 1.06	0.234	−0.064	0.935

CAI	Patience	10–70	33–70	61.07 ± 7.01	6.11 ± 0.70	−1.064	1.431	0.851
	Knowing	14–98	49–98	83.28 ± 11.21	5.95 ± 0.80	−0.646	−0.062	0.916
	Courage	13–91	13–87	46.44 ± 19.3	3.57 ± 1.48	−0.095	−0.809	0.940
	Total score	37–259	148–248	190.79 ± 21.47	5.16 ± 0.58	0.473	−0.347	0.851

NCCCS	Basic language communication competency	11–55	33–55	49.30 ± 5.58	4.48 ± 0.51	−0.840	0.189	0.937
	Emotional support competency	6–30	18–30	26.60 ± 3.51	4.43 ± 0.59	−0.629	−0.504	0.962
	Team communication competency	6–30	17–30	27.80 ± 3.02	4.63 ± 0.50	−1.346	1.405	0.950
	Communication competency in difficult clinical scenes	19–95	48–95	84.56 ± 11.07	4.45 ± 0.58	−0.802	−0.047	0.984
	Basic non‐language communication competency	7–35	21–35	31.80 ± 3.67	4.54 ± 0.52	−0.871	−0.006	0.951
	Emotional perception competency	9–45	23–45	40.26 ± 5.26	4.47 ± 0.58	−0.786	−0.136	0.979
	Total score	58–290	174–290	260.30 ± 30.61	4.49 ± 0.53	−0.826	0.015	0.992

JPS	Task performance	9–45	27–45	40.70 ± 4.78	4.52 ± 0.53	−0.758	−0.444	0.967
	Contextual performance	7–35	21–35	31.84 ± 3.72	4.55 ± 0.53	−0.799	−0.327	0.967
	Total score	16–80	48–80	72.54 ± 8.34	4.53 ± 0.52	−0.789	−0.322	0.981

*Note:* min, minimum score; max, maximum score.

Abbreviations: CAI, Caring Ability Inventory; ELS, Emotional Labor Scale; JPS, Job Performance Scale; NCCCS, Nurses’ Clinic Communication Competency Scale; SD, standard deviation.

The sum scores of the four key variables were (55.46 ± 14.79), (190.79 ± 21.47), (260.30 ± 30.61), and (72.54 ± 8.34). The reliability of all scales in this study was good.

### 3.4. Correlation Analysis

Pearson’s correlation analysis showed that ELS and its subscales had a statistically significant correlation with CAI (*p* < 0.001), with ELS showing a significant negative correlation with CAI (*r* = −0.273, *p* < 0.001). In the subdimensions of ELS, only deep acting had a statistically significant correlation with NCCCS and JPS (*r* = 0.408–0.425, *p* < 0.001). There was a significantly positive correlation between the variables CAI, NCCCS, and JPS (*r* = 0.374–0.874, *p* < 0.001), and the positive relationship between NCCCS and JPS was strong (*r* = 0.874, *p* < 0.001). The convergent validity and discriminant validity of all scales were good. The specific results are shown in Table 3.

**TABLE 3:** The correlation of emotional labor, caring ability, nurses’ clinic communication competency, and job performance (*N* = 210).

	**Surface acting**	**Emotional expression requirements**	**Deep acting**	**ELS**	**CAI**	**NCCCS**	**JPS**	**CR**	**AVE**	**AVE square root**

Surface acting	1							—	—	—
Emotional expression requirements	0.758^∗∗∗^	1						—	—	—
Deep acting	0.274^∗∗∗^	0.362^∗∗∗^	1					—	—	—
ELS	0.938^∗∗∗^	0.897^∗∗∗^	0.508^∗∗∗^	1				0.776	0.569	0.754
CAI	−0.389^∗∗∗^	−0.284^∗∗∗^	0.314^∗∗∗^	−0.273^∗∗∗^	1			0.645	0.591	0.769
NCCCS	−0.014	0.072	0.408^∗∗∗^	0.104	0.374^∗∗∗^	1		0.974	0.863	0.929
JPS	−0.032	0.033	0.425^∗∗∗^	0.082	0.422^∗∗∗^	0.874^∗∗∗^	1	0.962	0.927	0.963

Abbreviations: AVE, average variance extracted; CAI, Caring Ability Inventory; CR, composite reliability; JPS, Job Performance Scale; NCCCS, Nurses’ Clinic Communication Competency Scale.

^∗∗∗^ *p* < 0.001.

### 3.5. The Chain‐Mediation Effect Analysis

This study used a structural equation model to explore the relationship between variables and to investigate the mediating role of caring ability and communication competency. The structural equation consists of four latent variables: emotional labor (surface acting, emotional expression requirements, and deep acting), caring ability, communication competency, and job performance.

**FIGURE 1:**
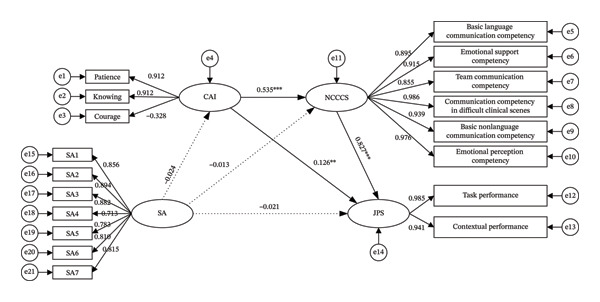
The chain‐mediation effect model diagram of surface acting, caring ability, communication competency, and job performance of emergency nurses (standardized coefficients in Model 1). Note: ^∗∗∗^ *p* < 0.001 and^∗∗^ *p* < 0.01. Abbreviations: SA: surface acting; CAI: Caring Ability Inventory; NCCCS: Nurses’ Clinic Communication Competency Scale; JPS: Job Performance Scale.

The mediation model between surface acting, caring ability, communication competency, and job performance (Model 1) is shown in Figure 1. The bootstrap method found that the bias‐corrected 95% confidence interval (95% CI) of the chain‐mediation path from surface acting to job performance was [−0.031, 0.025], which included 0, indicating the absence of the chain‐mediation effect (see Table 4).

**TABLE 4:** Bootstrap analysis of the significance test of mediation effects.

Effect	Pathway	Point estimate	Product of coefficients		Bootstrapping				Two‐tailed significance
					Percentile 95% CI		Bias‐corrected 95% CI		
			*SE*	*Z*	Lower	Upper	Lower	Upper	
Indirect effect	SA ⟶ CAI ⟶ JPS	−0.001	0.004	−0.250	−0.010	0.008	−0.012	0.007	0.625
	SA ⟶ NCCCS ⟶ JPS	−0.004	0.018	−0.222	−0.042	0.031	−0.043	0.030	0.777
	SA ⟶ CAI ⟶ NCCCS ⟶ JPS	−0.004	0.014	−0.286	−0.030	0.026	−0.031	0.025	0.735

Direct effect		−0.008	0.013	−0.615	−0.030	0.012	−0.029	0.014	0.516

Total effect		−0.017	0.025	−0.680	−0.066	0.031	−0.068	0.029	0.491

Abbreviations: CAI, Caring Ability Inventory; CI, confidence interval; JPS, Job Performance Scale; NCCCS, Nurses’ Clinic Communication Competency Scale; SA, surface acting.

**FIGURE 2:**
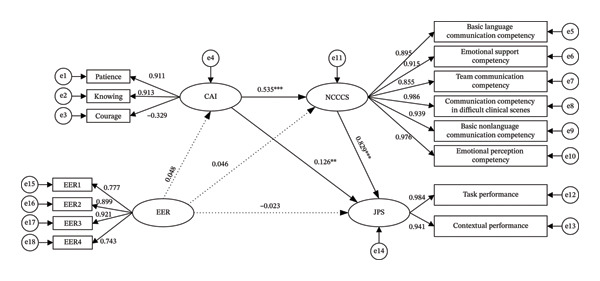
The chain‐mediation effect model diagram of emotional expression requirements, caring ability, communication competency, and job performance of emergency nurses (standardized coefficients in Model 2). Note: ^∗∗∗^ *p* < 0.001 and ^∗∗^ *p* < 0.01. Abbreviations: EER: emotional expression requirements; CAI: Caring Ability Inventory; NCCCS: Nurses’ Clinic Communication Competency Scale; JPS: Job Performance Scale.

The mediation model between emotional expression requirements, caring ability, communication competency, and job performance (Model 2) is shown in Figure 2. The bootstrap method found that the bias‐corrected 95% CI of the chain‐mediation path from emotional expression requirements to job performance was [−0.022, 0.044], which included 0, indicating the absence of the chain‐mediation effect (see Table 5).

**TABLE 5:** Bootstrap analysis of the significance test of mediation effects.

Effect	Pathway	Point estimate	Product of coefficients		Bootstrapping				Two‐tailed significance
					Percentile 95% CI		Bias‐corrected 95% CI		
			*SE*	*Z*	Lower	Upper	Lower	Upper	
Indirect effect	EER ⟶ CAI ⟶ JPS	0.003	0.005	0.600	−0.007	0.014	−0.006	0.016	0.466
	EER ⟶ NCCCS ⟶ JPS	0.016	0.021	0.762	−0.023	0.058	−0.023	0.058	0.402
	EER ⟶ CAI ⟶ NCCCS ⟶ JPS	0.009	0.017	0.529	−0.021	0.046	−0.022	0.044	0.609

Direct effect		−0.010	0.014	−0.714	−0.038	0.015	−0.037	0.015	0.453

Total effect		0.018	0.028	0.643	−0.035	0.077	−0.041	0.071	0.590

Abbreviations: CAI, Caring Ability Inventory; CI, confidence interval; EER, emotional expression requirements; JPS, Job Performance Scale; NCCCS, Nurses’ Clinic Communication Competency Scale.

**FIGURE 3:**
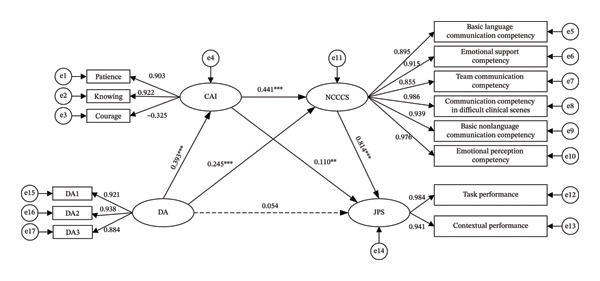
The chain‐mediation effect model diagram of deep acting, caring ability, communication competency, and job performance of emergency nurses (standardized coefficients in Model 3). Note: ^∗∗∗^ *p* < 0.001 and^∗∗^ *p* < 0.01. Abbreviations: DA: deep acting; CAI: Caring Ability Inventory; NCCCS: Nurses’ Clinic Communication Competency Scale; JPS: Job Performance Scale.

The mediation model between deep acting, caring ability, communication competency, and job performance (Model 3) is shown in Figure 3. The bootstrap method found that the bias‐corrected 95% CI of the chain‐mediation path from deep acting to job performance was [0.035, 0.120], which did not include 0, indicating the existence of the chain‐mediated effect (see Table 6), so we chose the results of Model 3 as the final model.

**TABLE 6:** Bootstrap analysis of the significance test of mediation effects.

Effect	Pathway	Point estimate	Product of coefficients		Bootstrapping				Two‐tailed significance
					Percentile 95% CI		Bias‐corrected 95% CI		
			*SE*	*Z*	Lower	Upper	Lower	Upper	
Indirect effect	DA ⟶ CAI ⟶ JPS	0.022	0.012	1.833	0.002	0.049	0.004	0.055	0.018
	DA ⟶ NCCCS ⟶ JPS	0.100	0.032	3.125	0.047	0.172	0.046	0.171	0.001
	DA ⟶ CAI ⟶ NCCCS ⟶ JPS	0.071	0.022	3.227	0.034	0.118	0.035	0.120	0.001

Direct effect		0.027	0.019	1.421	−0.005	0.064	−0.003	0.066	0.094

Total effect		0.219	0.045	4.867	0.136	0.319	0.129	0.309	0.002

Abbreviations: CAI, Caring Ability Inventory; CI, confidence interval; DA, deep acting; JPS, Job Performance Scale; NCCCS, Nurses’ Clinic Communication Competency Scale.

After modification, the fitting indexes of each model were relatively good: *χ* ^2^/df = 1.293, RMSEA = 0.037, GFI = 0.976, AGFI = 0.954, CFI = 0.994, IFI = 0.995, and NFI = 0.976. The total effect of deep acting on job performance was 0.219 (*p* < 0.05). However, the direct effect of the path was 0.027 (*p* > 0.05), and the result was insignificant. The mediating effect showed that the total indirect effect was composed of three paths, which suggested a complete mediating effect between deep acting and job performance. The three paths were as follows: the mediating effect of caring ability between deep acting and job performance was 0.022, the mediating effect of communication competency between deep acting and job performance was 0.100, and the chain‐mediating effect of caring ability and communication competency between deep acting and job performance was 0.071.

## 4. Discussion

The results of this study showed that the mean score of emergency nurses’ JPS score was 4.53 ± 0.52, which was higher than the results of Yu et al. [49]. This may be related to the JPS used in this study, which included some items such as “being able to take appropriate measures in emergencies,” “continuously monitoring changes in the patient’s condition,” and “proficiently using various equipment at work.” These items demonstrated abilities consistent with some essential skills of emergency nurses. Research indicates that emergency department nurses require good observational skills, trustworthy professional knowledge, and adaptable flexibility to effectively respond to diverse emergencies [50, 51], along with proficiency in various medical instruments and equipment [52] to guarantee efficient and safe utilization in critical situations. The ELS score was 3.96 ± 1.06, similar to Fang’s research on emergency department nurses [53] and higher than Zeng’s findings [54]. Furthermore, the scores for surface acting, emotional expression requirements, and deep acting surpassed those reported in Qiu’s study [15]. This may be related to the large number of emergency department patients and their conditions’ complexity and variability. While dealing with various emergencies, nurses need to constantly adjust their emotions to meet the needs of patients or their families, resulting in an increased application of emotional labor. In the three dimensions of ELS examined in this study, the average score of deep acting was the highest. In contrast, the average score of surface acting was the lowest, indicating that the survey participants were more inclined toward using deep acting as an emotion regulation strategy. The findings aligned with Wu’s survey of nurses across 92 hospitals in China [13], although they diverged from Hwang’s survey of nurses in general hospitals in Korea [55], which may be attributable to the distinct geographical and cultural backgrounds of the survey subjects. The NCCCS score was 4.49 ± 0.53, somewhat exceeding findings related to ICU nurses [56] and mental health nurses [16]. This may be attributed to the requirement for emergency department nurses to possess specific communication competencies to handle emergencies and collaborate smoothly with medical teams. However, the CAI score of emergency department nurses in this study was relatively low, measuring only 190.79 ± 21.47, which was inferior to the findings of He’s study [57]. The quick turnover and intense work pressure in the emergency department hinder nurses from patiently listening to patients’ needs, paying attention to their emotional changes, and delivering essential psychological support and comfort.

This study indicated that among the dimensions of emotional labor, only deep acting correlated with job performance. On the contrary, the other two dimensions and the overall emotional labor did not relate to job performance. More excellent deep acting is associated with improved performance in emergency department nurses. Deep acting is a positive emotional regulation strategy in which an individual adjusts their emotional state through active cognition, imaginative engagement, and recollection [14]. Deep acting means that nurses endeavor to modify their feelings to correspond with the emotional expressions mandated by their profession. This strategy enables nurses to sustain high focus and engagement in their roles, thus boosting work efficiency and patient satisfaction, ultimately improving job performance [58]. More importantly, nurses can convey their emotions more genuinely through deep acting, thus developing a trusting relationship with patients and enhancing the quality of nursing care. When patients perceive the sincerity and caring of nurses, they are more likely to engage cooperatively in their treatment, hence facilitating the efficient advancement of nursing tasks.

This study examined the correlation between three emotional labor strategies and job performance, emphasizing the role played by caring ability and communication competence. The initial verified pathway was the complete mediating role of caring ability between deep acting and job performance. This discovery highlighted the significant influence of individual inner emotional regulation on caring ability. Nurses’ primary duty in emotional labor is to exhibit caring [59]. The surface acting strategy masks real emotions and contributes to fatigue, emotional exhaustion, stress, and withdrawal from work; conversely, deep acting can promote communication with patients, job satisfaction, organizational commitment, and caring behavior [15, 60]. When emergency department nurses regulate their emotions to fulfill job demands through deep acting strategy, they are better able to empathize with patients’ needs and exhibit more remarkable caring ability, which directly promotes job performance, as caring behavior can increase patients’ trust and satisfaction, consequently elevating nursing quality and overall work effectiveness.

This study also confirmed that communication competence fully mediated the relationship between deep acting and job performance. Previous studies have shown that nurses actively adjust their inner emotions and stimulate empathy through deep acting, establishing trusting relationships with patients and promoting nurse–patient communication [61]. Effective communication can promote nurses’ prosocial behaviors and raise nursing performance and quality [62]. This finding revealed the beneficial impact of deep acting on communication competence. Emergency department nurses enhanced their emotional regulation, communication sensitivity, and proficiency by employing deep acting. They can express their feelings and intentions more effectively and mitigate misunderstandings and disputes, fostering harmonious and successful workplace interactions. Enhancing this communication competence directly facilitates information exchange and resource integration, establishing a basis for improved job performance.

In addition, our research findings demonstrated that caring ability and communication competence had a chain‐mediated effect on deep acting and job performance. This study found that deep acting did not directly influence job performance; instead, it indirectly impacted job performance through two mediating variables: caring ability and communication competence. Prior research has also indicated that nurses utilizing deep acting tactics can more easily identify and regulate their emotions, exhibit greater empathy, treat patients sincerely, and communicate effectively, enhancing job performance [49]. Specifically, emergency department nurses are better prepared to recognize and care for patient needs by modulating their genuine emotions following expression norms, thereby improving their caring ability. Such caring behavior is frequently associated with more transparent and authentic communication, fostering effective interactions among emergency department nurses, patients, and teams. It ultimately has a positive impact on job performance.

## 5. Limitations

The limitations of this study were as follows: first of all, the cross‐sectional methodology overlooked temporal dynamics and causal linkages, and future longitudinal studies should be undertaken to explore the trajectory changes of these variables. Second, the sample size was relatively limited and failed to represent the entirety of Chinese emergency department nurses. Caring ability and communication competence were not identified as mediators in the relationship between the other two dimensions of emotional labor (surface acting and emotional expression requirements) and job performance. Increasing the sample size to verify this link is essential in the future. Third, 83.3% of the participants in this study were female, potentially indicating unaccounted gender disparities that were not reflected in the research results. Fourth, the study used a convenience sample method, which may cause selection bias. In the future, random stratified sampling approaches should be utilized to recruit participants. Finally, all variables in this study were assessed by self‐report, which may bring in reporting bias. In the future, tools may be created to objectively measure nurses’ caring and communication behaviors in clinical environments.

## 6. Conclusions

In summary, the hypothesis in this study was partially valid. Emotional labor did not directly influence job performance. The emotional management strategy of deep acting was the sole approach that indirectly influenced job performance via the mediating role of caring ability and communication competence. Conversely, the other two strategies (surface acting and emotional expression requirements) did not exhibit a significant impact. Therefore, nursing managers and educators should attach importance to the emotional labor of emergency department nurses, particularly the cultivation and fostering of deep acting. Nurses’ deep acting can be developed by offering emotional cognition and expression training, simulating situational exercises, establishing incentive mechanisms, enhancing communication skills and humanistic care education, promoting interdepartmental collaboration, and other measures. In addition, their work motivation can be invigorated, and their caring ability and communication competency can be improved to provide better nursing services to patients.

## Funding

This research was funded by the National Natural Science Foundation of China (Project 71874063) and the Research Fund Nursing Special Project of Tongji Hospital (2023D48 and 2024D08).

## Ethics Statement

The study was conducted in accordance with the Declaration of Helsinki and was approved by the Medical Ethics Committee of Tongji Hospital Affiliated to Tongji Medical College of Huazhong University of Science and Technology, China (approval number: TJ‐IRB202401086).

## Conflicts of Interest

The authors declare no conflicts of interest.

## Data Availability

The data that support the findings of this study are available from the corresponding author upon reasonable request.
